# Open Set Audio Classification Using Autoencoders Trained on Few Data

**DOI:** 10.3390/s20133741

**Published:** 2020-07-03

**Authors:** Javier Naranjo-Alcazar, Sergi Perez-Castanos, Pedro Zuccarello, Fabio Antonacci, Maximo Cobos

**Affiliations:** 1 Visualfy, 46181 Benisanó, Spain; sergi.perez@visualfy.com (S.P.-C.); pedro.zuccarello@visualfy.com (P.Z.); 2 Computer Science Department, Universitat de València, 46100 Burjassot, Spain; maximo.cobos@uv.es; 3 Dipartimento di Elettronica, Informazione e Bioingegneria (DEIB), Politecnico di Milano, 20133 Milan, Italy; fabio.antonacci@polimi.it

**Keywords:** open set recognition, open set classification, audio classification, autoencoders, few-shot learning

## Abstract

Open-set recognition (OSR) is a challenging machine learning problem that appears when classifiers are faced with test instances from classes not seen during training. It can be summarized as the problem of correctly identifying instances from a known class (seen during training) while rejecting any unknown or unwanted samples (those belonging to unseen classes). Another problem arising in practical scenarios is few-shot learning (FSL), which appears when there is no availability of a large number of positive samples for training a recognition system. Taking these two limitations into account, a new dataset for OSR and FSL for audio data was recently released to promote research on solutions aimed at addressing both limitations. This paper proposes an audio OSR/FSL system divided into three steps: a high-level audio representation, feature embedding using two different autoencoder architectures and a multi-layer perceptron (MLP) trained on latent space representations to detect known classes and reject unwanted ones. An extensive set of experiments is carried out considering multiple combinations of openness factors (OSR condition) and number of shots (FSL condition), showing the validity of the proposed approach and confirming superior performance with respect to a baseline system based on transfer learning.

## 1. Introduction

Machine listening is the branch of artificial intelligence that aims to create intelligent systems that are capable of extracting relevant information from audio data. Acoustic event classification (AEC) and acoustic scene classification (ASC) are two areas that have grown significantly in the last years [1,2,3,4], often included within the machine listening field. The increase in research proposals related to these areas is motivated by the number of applications that can benefit from automation systems incorporating audio-based solutions, such as home assistants or autonomous driving. This interest is also evidenced by the multiple editions of the successful international DCASE challenge (Detection and Classification of Acoustic Scenes and Events). From its very first edition in 2013 [5], different ASC and AEC tasks have been presented during the past years (2013, 2016, 2017 and 2018). In fact, the 2019 edition incorporated an open-set recognition (OSR) task within the scope of ASC, where the idea was to classify an audio clip to a known scene type or to reject it when it belonged to an unknown scene.

In general terms, OSR is a problem that appears when an intelligent system has to classify (in inference stage) a sample from an unknown class, i.e., a class that has not been seen during training. The complexity of the OSR problem can be quantified by using the openness factor ($O^{*}$) presented in [6], which measures the relationship between the number of classes seen during training and the number of classes seen during the inference stage only. The objective of a system that is deployed to face an OSR environment is to classify correctly the samples that belong to classes that have been seen during training, while properly rejecting samples from unknown classes. The most popular solutions aimed at solving OSR problems make use of classic machine learning algorithms such as support vector machines [7] or nearest neighbors [8]. In this context, deep learning solutions are not so common in this problem, showing the need for further investigation in this direction [9,10].

Few-shot learning (FSL) is another phenomenon related to real-world applications that aims to detect a specific pattern or class with little amount of data for training the classification system, i.e., using few examples per class. FSL has been widely investigated in face recognition tasks. However, contributions in the audio domain are not so common and are mostly related to music fraud detection [11] or speaker identification [12,13]. A main feature of FSL has to do with the “intra-class” behavior of coarse categories. As an example, assume that a general class “bell” groups samples from different types of bells. The goal of FSL would be to discern among the different bell types, even if all of them can be categorized into a general “bell” class. Two different approaches can be followed to tackle FSL. On the one hand, the transfer learning (TL) approach [14] tries to solve the problem of having only few samples by using prior knowledge. This prior-knowledge is usually represented by the use of a neural network pre-trained on external data that is employed as a feature extractor [15]. The other approach lies on novel neural network architectures such as Siamese [16,17], facenet (trained with triplets) [18,19] or on classical networks trained with novel loss functions such as ring loss [20] or center loss [21]. The main problem with these networks is that a relatively large amount of data is required to properly generalize FSL tasks, i.e., the need to consider many different classes even if only few samples are available per each class.

Recently, a dataset that takes into account both limitations (OSR and FSL) has been made public by the authors [22]. This dataset is composed by two coarse classes: pattern and unwanted sounds. The pattern sounds class is made up of 24 subclasses. These subclasses correspond to specific patterns of different domestic alarms such as bells or fire alarms. Therefore, all these 24 subclasses can be considered as a coarse, more general, “domestic alarm” class, but providing intra-class differentiation within it. On the other hand, the unwanted sounds are grouped into 10 different subclasses with more general and likely to appear domestic sounds, such as keyboard tapping, cough or music among others. These samples must be rejected by an OSR classification algorithm. All these subclasses, either pattern and unwanted, contain 40 samples. The dataset is provided with different configurations depending on the openness factor or the number of shots. In turn, these configurations are divided into different k-fold configurations depending on the number of training examples to facilitate the analysis of the generalization of the proposed solutions.

This paper proposes a novel deep learning approach to tackle OSR and FSL problems within an AEC context, based on a combined two-stage method. As a first step, an embedded or bottleneck representation from the audio log-Mel spectrogram is obtained by means of an autoencoder architecture. Once the autoencoder is trained, the bottleneck representation is used to train a simple multi-layer perceptron (MLP) classifier with sigmoid activation for OSR classification. The autoencoder part aims at solving the FSL limitation, while the MLP classifier mitigates the OSR problem. Moreover, two autoencoder alternatives are suggested within the considered framework, considering both semi-supervised and unsupervised training. Thus, the contributions of this paper reside on the proposal of a full framework for AEC OSR/FSL tasks, the analysis of this framework in different OSR/FSL conditions (different openness values and number of training samples) and the comparison with the baseline method presented in the dataset release [22], showing significant improvement without the need to use prior knowledge from external data.

The rest of the paper is organized as follows. The required background describing OSR openness and autoencoders can be found in Section 2. The proposed system and its different parts are presented in Section 3. The experimental details, including datasets and parameter configuration are described in Section 4, while the results are discussed in Section 5. Finally, conclusions and future work are summarized in Section 6.

## 2. Background

This section reviews the background and previous works related to the proposed framework, including FSL, OSR and the use of autoencoders in audio-related tasks.

### 2.1. Few-Shot Learning

Few-shot learning (FSL) is the problem that appears in machine learning applications when a small amount of data is provided per class. In fact, machine learning techniques have become state-of-the-art solutions in many domains due to the huge data sets available.

The FSL limitation can be addressed in three different strategies according to [23]. The three possible approaches are: modifying the available data (increasing the training data), choosing a particular model with FSL considerations during the training and testing stages or using prior knowledge solutions. In this work, FSL was approached by the creation of a specific model making use of autoencoders. Within the strategy of creating specific models for FSL there are, in turn, different approximations. In our particular case, the use of autoencoders represents a solution based on embedding learning, that is, a model that is capable of discovering important structure within the input data by forcing a reduction of dimensionality. For a complete review of FSL approaches, the reader is referred to [23].

One of the first appearances of an architecture to solve an FSL task with an embedding learning approach was in signature recognition. The proposed architecture is known as the Siamese network [16]. The main feature of a framework based on Siamese networks is that it instances two networks having the same architecture and tied weights, forcing the network to learn the similarities between the two inputs. The purpose of this framework is to train a network that is able to embed the inputs into a domain having lower dimensionality in a smart way. That means that if the two entries are very similar, the embeddings must be similar. Once the network is trained, two entries are passed through the network and a measurement metric is calculated to determine if both entries are in the same class.

Triplet networks appeared as a modification of Siamese networks [18]. In a similar way, the framework is created by instantiating three networks with tied weights. In each step, the network is fed with one example called anchor, one positive and one negative. The positive sample has to belong to the same class as the anchor and the negative one to a different class. In both cases (Siamese or triplet networks), the selection of pairs or triplets is crucial for an efficient training process.

A different approach to address the FSL issue is to modify the network loss function to emphasize the distance between classes in the feature map space during training [24]. Some examples are ring loss [20], center loss [21] or prototypical networks [25]. Ring loss and center loss can be understood as a modified softmax that tries to obtain more discriminative features with a modification during loss calculation. The objective of prototypical networks is to obtain a cluster center for each class. During the inference stage, the classification is carried out by using the distances to each center.

The above solutions have shown promising results in the field of image and computer vision. Note, however, that although there are few samples per class, the datasets are considerably large. For example, in [26], there are about 13,500 examples in total. This number of examples might be enough to train the above kind of solutions. However, as far as this group is concerned, in the audio domain, there are not FSL datasets with such amount of data. As a result, the proposed autoencoder-based approach accommodates better the scenario considered in this work.

### 2.2. Open-Set Recognition

In realistic scenarios there is usually an incomplete knowledge of all the possible surrounding classes at the time of training, and a trained classifier may face unknown classes during testing. As a result, algorithms need to accurately classify the known classes, but also to deal effectively with the unknown ones. OSR approaches are designed to do both things properly. When dealing with OSR problems, certain considerations should be kept in mind when defining which classes are to be recognized and which should be rejected. The evaluation of OSR systems is based on the concept of openness factor $O^{*}$ [6], which introduces a categorization on the classes involved in the training and testing stages:

- *Known Known* (KK) classes: classes that are used in the training and validation stage and that must be correctly classified by the system.
- *Known Unknown* (KU) classes: classes that are available during the training stage but must not be categorized into the specific class they belong to. In other words, they must be rejected by the classifier. These classes are very useful since they allow the system to make representations and generate boundaries that can help to discern samples from the unwanted category.
- *Unknown Known* (UK) classes: classes for which no samples are available during training but side-information such as semantic/attribute information is available during training. This category is not considered in this work.
- *Unknown Unknown* (UU) classes: classes that are not used nor in the training nor in the validation stage and must obviously be rejected by the classifier. The system only sees these classes in the test stage.

According to [27], the openness factor is defined as

(1) $$O^{*}=1-\sqrt{\frac{2\times T_{TR}}{T_{TR}+T_{TE}}}\;,$$

where $T_{TR}$ corresponds to the total number of classes used in the training stage (either KK or KU) and $T_{TE}$ corresponds to the number of classes used in inference stage. When $O^{*}=0$, $T_{TR}=T_{TE}$, meaning that there is no UU class. On the other hand, when $T_{TE}$ becomes larger and $T_{TE}>T_{TR}$, $O^{*}\overset{}{\to }1$, leading to a more complex OSR task. Note that, by definition, the openness factor is bounded to the range $0\leq O^{*}<1$.

Different approaches have been taken to address the issue of OSR, either with discriminative models or generative models. Traditional machine learning frameworks have been used as enhanced discriminatory models, such as those based on SVM solutions, including the Weibull-calibrated SVM (W-SVM) [28] or P_I_-SVM [29]. Other approaches based on classic techniques are those based on sparse representation (SROSR) [30]. As reported in the original paper, the training set must be large enough to cover the conditions that may be present in the test stage. Distance-based methods with modifications have also been proposed [8,27,31,32].

With regard to deep-learning-based solutions, there is the problem of their original close-set nature. The first approach to create deep neural networks of open-set nature was to replace the commonly used final Softmax layer with an OpenMax layer [9]. Other approaches are the deep open classifier (DOC) [33] or the competitive overcomplete output layer (COOL) [34]. More solutions provided in the context of DNN are discussed in [27].

While all the these approaches have shown to improve classification systems in OSR conditions, they also have their limitations [27]. One of the main problems is that the classifier is not able to understand the whole context when dealing with unknown classes. The framework presented in this paper relies on the latent space distribution learned by autoencoders, which is assumed to compact the information from the training classes into a space that can be more easily handled by a subsequent decision stage. As it will be explained in Section 3, a DNN with sigmoid activation will be used for this task.

### 2.3. Autoencoders in Audio Processing Tasks

The autoencoder is a machine learning solution made up of two blocks, encoder and decoder, whose purpose is to obtain internal representations usually with smaller dimensionality than the input. This process is known as encoding. For this representation to be obtained, the decoding phase is also necessary so that the system can encode efficiently the input data. The purpose of this block of the autoencoder is to reconstruct the input signal from the intermediate representation obtained by the encoder. The difference between the reconstructed signal by the autoencoder and the original input signal is known as the reconstruction error. In essence, the autoencoder tries to learn an identity function h(x)≈x, which makes the output $\hat{x}$ be similar to the input x. By placing constraints on the network, such a limitation in the number of hidden units, interesting structure about the data can be discovered. Although there are different types of autoencoders (e.g., vanilla multi-layer autoencoders, denoising autoencoders, convolutional autoencoders or variational autoencoders) the underlying fundamental principle is the same. For example, convolutional autoencoders are designed to encode the input into a set of simpler signals and reconstruct the input from them. The encoder layers are in this case convolutional layers and the decoder layers are called deconvolution or upsampling layers.

In the audio domain, autoencoders have become the state-of-the-art solution for speech translation applications [35]. Besides, other tasks such as learning more sophisticated or universal audio representations or anomalous sound detection currently tend to solve their limitations using autoencoders. The following paragraphs describe some previous work in this direction, where autoencoders are used to solve the aforementioned problems.

In [36,37], different autoencoder architectures and approaches were presented to obtain robust audio representations that can be used in a variety of audio tasks. In [36], audio representations are learned by addressing a phase prediction task. The autoencoder in [37] was trained in an unsupervised way using Audioset [38], one of the largest audio datasets. In this case, the autoencoder was implemented with convolutional layers. The experimental work is performed considering small encoder architectures that can be potentially deployed on mobile devices.

Another interesting audio application of autoencoders is anomalous sound detection, which is the task of identifying whether a sound corresponds to a normal (known) or abnormal (unwanted) class [39,40]. The main challenge of this problem is to detect the anomaly having only training samples of normal behavior. The objective can be to detect machine faults only by monitoring the sound produced by these machines. The mean squared error obtained when reconstructing the signal can provide information on whether the sample is normal or abnormal.

The approach presented in this work uses an autoencoder in order to obtain discriminative intra-class audio representations. The use of autoencoders to discriminate unwanted classes has already been suggested in the literature. For example, in [41], a solution to detect known or unwanted scenes is presented. In this method, an autoencoder is trained for each known class and the reconstruction error is used to decide if the class is known or not. In contrast, our proposal considers a single autoencoder trained on all the known classes (KK) and the intermediate layer or bottleneck is used to train a MLP to distinguish unwanted samples.

## 3. Proposed Approach

**Figure 1:**
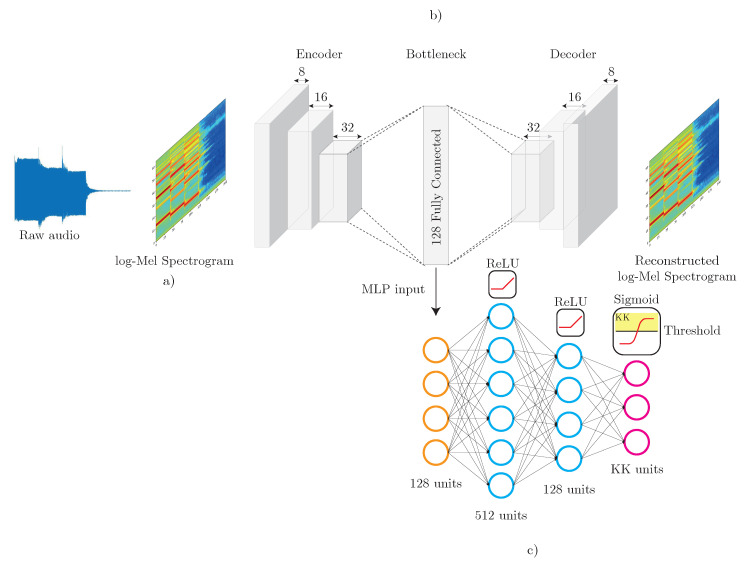
Proposed open-set recognition (OSR)/few-shot learning (FSL) framework for audio classification. In this scheme, an unsupervised autoencoder is considered. (**a**) Log-Mel spectrogram representation. (**b**) Autoencoder. (**c**) multi-layer perceptron (MLP) classifier.

This section presents the proposed solution to address the problems of FSL and OSR jointly, which consists of three blocks: a high-level 2D time-frequency audio representation, a smaller dimensional encoding of such representation using an autoencoder and a final MLP classifier aimed at discerning whether the input corresponds to a known class or to an unknown class. The full framework is depicted in Figure 1.

### 3.1. Input Audio Representation

To facilitate learning from few data, the raw audio input is first transformed into a meaningful time-frequency audio representation. A state-of-the-art choice for many audio processing tasks is the use of log-Mel spectrograms [3,42]. This representation is calculated with a window size of 40 ms and an 50% overlap. The number of Mel filters is set to 64. Each frequency bin is normalized to zero mean and unit standard deviation using all the available training data.

### 3.2. Convolutional Autoencoder

**Figure 2:**
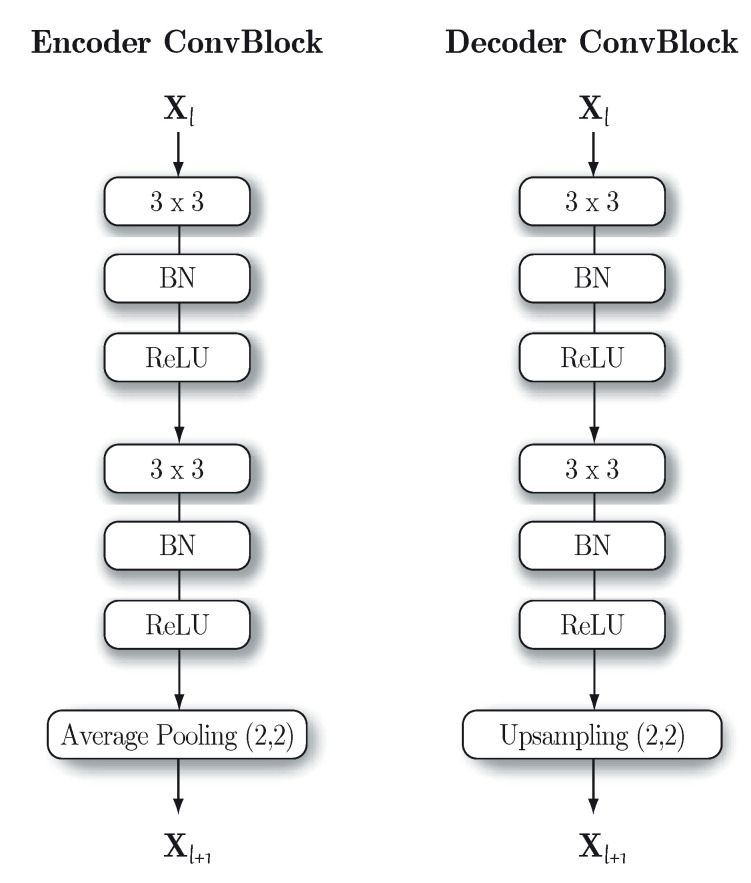
Architecture of the ConvBlocks for the two parts of the autoencoder. $X_{l}$ denotes the input to the block, while $X_{l+1}$ denotes the output.

The proposed system considers the use of a convolutional autoencoder made up of convolutional layers. In this work, a convolutional block is understood to consist of a convolutional layer, a batch normalization layer (BN) and a non-linear activation, in our case rectified linear units (ReLU). This same configuration is stacked again with an increasing number of filters and ends with an average pooling layer (2,2) [43]. Therefore, each convolutional block (ConvBlock) is made up of seven layers (see Figure 2). The decoder follows a symmetric structure with respect to the encoder, that is, the number of filters in each decoding ConvBlock decreases until reaching the last convolutional layer, which has only a single filter and is in charge of obtaining the reconstructed input. Another consideration is that average pooling layers are replaced by upsampling layers. The ConvBlock architecture can be seen in Figure 2. The last convolutional layer is not accompanied by a normalization or activation layer. The only layers with linear activation are the last convolutional layer of the decoder and the bottleneck layer that corresponds to a dense layer. This dense layer acts as a representation of the encoded audio and is made up of 128 neurons. To prevent the autoencoder from learning the identity function, a dropout layer is introduced at the start of the encoder [44,45]. The autoencoder architecture is detailed in Table 1.

#### 3.2.1. Unsupervised Autoencoder

Autoencoders originally appeared as a solution to unsupervised problems [46], where no information about the class to which each sample belonged was available. Autoencoders can not be used for supervised classification problems and audio labels are not used in this stage of the training. The objective of the autoencoder is only to extract meaningful internal representations of each audio independently, leading to similar audio representations for samples belonging to the same class. In this case, the loss function during training corresponds to the mean squared error (MSE):

(2) $$L_{mse}=\frac{1}{N}\sum_{i=1}^{N}{\left(X_{i}-{\hat{X}}_{i}\right)}^{2}$$

where $X_{i}$ corresponds to an original log-Mel spectrogram and ${\hat{X}}_{i}$ to the one reconstructed by the autoencoder. Finally, *N* represents the number of samples in the batch.

#### 3.2.2. Semi-Supervised Autoencoder

**Figure 3:**
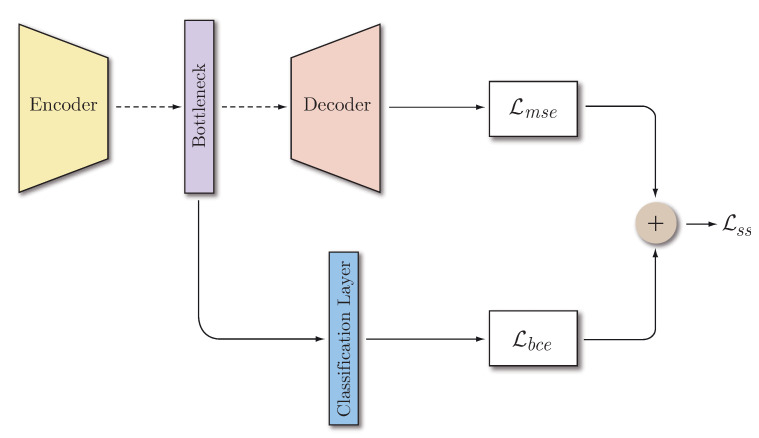
Semi-supervised autoencoder architecture.

In order to mitigate the assumption that samples of the same class have a similar representation and try to achieve more similar representations within the same intra-class, the autoencoder has been modified so that it not only takes into account the reconstruction error but also the classification error. The goal is to force the encoder to approximate representations of the same class in the feature space. Therefore, the bottleneck layer is stacked with a classification layer, a dense layer with the number of neurons equal to the number of KK classes. A block diagram of this architecture can be found in Figure 3. In this case, the total loss is a weighted sum of the reconstruction error (MSE) and the classification error, which in our case is the binary cross-entropy (BCE):

(3) $$L_{bce}=-\frac{1}{N}\sum_{i=1}^{N}\left[y_{i}\log{\hat{y}}_{i}+\left(1-y_{i}\right)\log\left(1-{\hat{y}}_{i}\right)\right]$$

(4) $$L_{ss}=\frac{1}{2}L_{mse}+\frac{1}{2}L_{bce}$$

where $y_{i}$ corresponds to the label of the original class instance and ${\hat{y}}_{i}$ to the predicted label. $L_{ss}$ is the loss used in the semi-supervised configuration. Each partial loss is multiplied by 1/2 to use a uniform weighting between reconstruction and classification. The choice of these weights is designed so that the framework is purely semi-supervised. If more weight is given to the reconstruction error, the framework will be more likely to appear to be the unsupervised system. On the other hand, if more weight is given to the classification error, the autoencoder will be more likely to address a closed-set classification problem and the open-set consideration will not be properly handled.

### 3.3. Multi-Layer Perceptron

Once the autoencoder has been trained, a MLP is trained on the learned latent space representations. This block will be in charge of classifying a sample if it belongs to a KK class (pattern category) or rejecting it either if it belongs to KU or UU classes (unwanted category). The MLP is trained with the representations that are obtained in the bottleneck layer of the autoencoder. Each audio sample is represented by 128 features. The MLP consists of three layers. The first two layers have 512 and 128 units respectively with ReLU activation. The last layer has as many units as KK classes and its activation is a sigmoid function. The output of the sigmoid is used as a likelihood score, so that a threshold is established for deciding if a given audio sample belongs to a KK class. This threshold is set to 0.5. If none of the outputs corresponding to the KK classes is above the threshold, the sample will be rejected. This MLP architecture is inspired in previous works, such as [15] or the baseline method used in [22]. This also allows for emphasizing the contribution of the proposed autoencoders, verifying their validity in a clearer way. In this context, the baseline method proposed in [22] uses audio embeddings obtained from the L3net network [15]. As a result, while the baseline employs transfer learning (the method relies on prior knowledge from a pre-trained network), we only make use of the samples available in the training dataset. Training details will be presented in Section 4.2.

## 4. Materials and Methods

This section describes the experimental framework considered in this paper, including the dataset used, the FSL/OSR conditions, the performance metrics considered or the network training details. Note that the proposed method is intended to address both the problem of OSR and the FSL problem. State-of-the-art solutions for such learning problems may not be suitable when both problems appear simultaneously. Therefore, special care must be taken when comparing systems not specifically designed to tackle both aspects.

### 4.1. Dataset

The dataset used to validate the framework proposed in this paper was recently presented in [22]. The dataset contains audio samples of a domestic nature to address FSL audio event recognition in an OSR context. The data set consists of two general classes or coarse categories defined as:

- *Pattern sounds category*: includes all the classes that must be recognized. Samples belonging to one of these classes must be classified as such. In our scenario, this category is made up of KK classes.
- *Unwanted category*: includes all the classes that should not be classified. Samples from these classes must be rejected by the system without labeling them. In this context, the unwanted category consists of the KU and UU classes, depending on the openness configuration.

The pattern category contains 24 classes that correspond to different domestic alarms and the unwanted category contains 10 classes of different nature such as cough, keyboard tapping or door slam among others. The dataset comes prepared for different openness values and different shots during training. As can be seen from Equation (1), the openness condition is affected by the number of KK classes to be classified or the number of unwanted classes used for training. Therefore, two approaches are presented within the dataset. In the first approach, the system is trained to recognize the full set of pattern classes (24 KK classes). As far as the unwanted classes are concerned, the dataset is designed so that the system is trained using all, half or no unwanted classes, leading to the set of openness values $O^{*}\in \left\{0,0.04,0.09\right\}$, respectively. Another scenario is the creation of known trios, i.e., only 3 classes of KK are used for training/testing. With this configuration 8 different training trios are created. By repeating the same process with respect to the unwanted ones, the resulting openness values are $0^{*}\in \left\{0,0.13,0.39\right\}$ with this configurations. The experiments that make up the configuration $O^{*}=0.39$ were not carried out because it was not possible to get a feasible solution with such an openness factor. The number of classes used during the training and inference stages that correspond to the openness values previously explained are specified in Table 2.

On the other hand, all these settings can be trained with different shots. The dataset was pre-configured for 4, 2 or 1-shot training. The number of shots modifies the k-fold cross-validation. For example, when training with 4 shots a 10-fold configuration was used, while when training with 1 shot, a 40-fold configuration was employed.

### 4.2. Training Procedure

The training setup was very similar for the autoencoder and the MLP. The optimizer used was Adam and the batch size was set to 32 samples. The learning rate starts with an initial value of 0.001 and decreases when the validation metric had not improved for 20 epochs by a factor of 0.75. The training was terminated if the validation metric did not improve for 50 epochs. The final selection corresponds to the model that had obtained a better metric in validation. The difference was the maximum number of epochs yet for the autoencoder is 500 epochs and for the MLP is 200 epochs. It must be here considered that when training from the scratch with few samples the system can easily converge to different local minima depending on its initialization. Therefore, each k-fold configuration was also repeated 5 times in order to provide insight about its statistical behavior and robustness.

### 4.3. Performance Metrics

The metrics used to analyze the performance of the proposed systems are presented in [22] and summarized here for the convenience of the reader. They are based on the weighted global accuracy, $ACC_{w}$, which is computed differently depending on the value of openness.

(5a) $$\begin{array}{c} \begin{array}{cc} O^{*}=0 & \;\left(\mathrm{without}\mathrm{UU}\right):\\ \; & ACC_{w}=wACC_{KK}+\left(1-w\right)ACC_{KU}, \end{array} \end{array}$$

(5b) $$\begin{array}{c} \begin{array}{cc} O^{*}\neq 0 & \;\left(\mathrm{with}\mathrm{KU}\mathrm{and}\mathrm{UU}\right):\\ \; & ACC_{w}=wACC_{KK}+\left(1-w\right)ACC_{KUU}, \end{array} \end{array}$$

(5c) $$\begin{array}{c} \begin{array}{cc} O^{*}\neq 0 & \;\left(\mathrm{with}\mathrm{only}\mathrm{UU}\right):\\ \; & ACC_{w}=wACC_{KK}+\left(1-w\right)ACC_{UU}, \end{array} \end{array}$$

where *w* represents a factor that weights the accuracy between the accuracy obtained over KK classes and other unwanted classes (KU or UU). $ACC_{KK}$ corresponds to the accuracy with which the system correctly classifies the KK samples into their respective classes. In this case, to accept a sample as a valid KK class, the OSR threshold (0.5) must be exceeded.

The performance metrics involving the unwanted category ($ACC_{KU}$, $ACC_{KUU}$ or $ACC_{UU}$), indicate the ability of the system to reject samples that do not belong to any KK class. For a sample to be considered unwanted, none of the KK classes must exceed the OSR threshold. The reason why there are three different metrics relates to the different openness conditions $O^{*}$. When the system sees all possible unwanted classes during training, the unwanted category only consists of KU classes. On the other extreme, if the system does not see any unwanted samples during training, the unwanted category is only made up of UU classes. When the system sees some of the classes pertaining to the unwanted category, the $ACC_{KUU}$ metric is used, which is defined as the average of $ACC_{KU}$ and $ACC_{UU}$.

## 5. Results and Discussion

This section discusses the results obtained for the different FSL/OSR configurations considered in the described dataset. The performance of the two proposed autoencoder-based systems is compared to the one obtained by the dataset baseline system. More detailed information about the number of classes used in each experiment can be seen in Table 2.

### 5.1. Full Set (24 KK) Performance

All the results of this configuration can be seen in Table 3. In the one-shot case, a substantial improvement over the baseline system for all the openness cases can be observed, with our two proposed approaches achieving a considerably higher $ACC_{w}$ value. The lowest improvement is of 7 percentage points ($O^{*}=0.04$), while the highest is almost of 30 percentage points ($O^{*}=0$). With this number of shots, both the $ACC_{KK}$ (in all cases) or the $ACC_{UU}$ (in $O^{*}=0.09$) are greatly improved. As observed, the baseline is very likely to classify the pattern sounds (KK classes) into the unwanted category, except when $O^{*}\neq 0.09$. Using autoencoders, more reliable representations are obtained for the KK classes, resulting in improved accuracy (see $ACC_{KK}$ and $O^{*}=0$). Something similar occurs for $ACC_{UU}$, where autoencoders can get more distant representations within the latent space for unwanted classes. All the metrics remain very similar for the unwanted category when $O^{*}\in \left\{0,0.04\right\}$.

The behavior is very similar when the number of training samples is 2 or 4. When $O^{*}\in \left\{0,0.04\right\}$ most metrics are improved to a greater or lesser extent. Only the unsupervised autoencoder shows worse behavior on the $ACC_{KUU}$ metric although it improves $ACC_{UU}$. However, the most significant contribution of the proposed frameworks can be seen when $O^{*}=0.09$. Just like for the one-shot case, $ACC_{UU}$ is clearly enhanced with this framework. In this case, the $ACC_{UU}$ is considerably improved with respect to the baseline, going from 33.3% to 72.5% (semi-supervised) and 26.1% to 69.9% (unsupervised).

Another factor to be analyzed is the standard deviation. That is to say, how the generalization of the solution is affected by the fact that few training samples are available. Depending on the initialization of the network, the results may differ. When a large data set is available, this fact is usually not very decisive. This is not the case in an FSL context. If the standard deviation is analyzed, it must be done taking into account the number of shots and the corresponding openness factor. Analyzing the KK classes, when $O^{*}=0$ it can be seen how the framework with the semi-supervised autoencoder has the lowest deviation in all possible cases depending on the number of shots. The unsupervised one has a higher standard deviation than the baseline when the number of shots is 2 or 4. This may be due to the fact that even though it is trained with more samples of the same class, the framework is not aware of it since it does not have such an information. Also, when $O^{*}\neq 0$ the standard deviation of the unsupervised is higher than the baseline if the number of shots is greater than 1. Probably, the system is becoming more prone to false negatives as the value of openness increases. When the framework does have information about the sample class (semi-supervised architecture), and the number of shots is bigger than one, the standard deviation is reduced. When $O^{*}>0$ and the number of shots is higher than one, the semi-supervised architecture has lower standard deviation than the baseline. This does not happen when the number of shots is equal to one since in this case the unsupervised has the lowest deviation. Regarding the unwanted category, it can be observed how deviations increase for all the methods as the value of openness increases. In this case, the framework with the semi-supervised autoencoder shows better results than the unsupervised one except for a single case with $ACC_{UU}$ and $O^{*}=0.04$. The reduction in standard deviation is much greater as the number of shots is increased, as seem for $ACC_{UU}$ when $O^{*}\in \left\{0.04,0.09\right\}$.

### 5.2. Trio Performance

The results obtained with this configuration are presented in Table 4 and Table 5. By looking first at the results in Table 4 ($O^{*}=0$), it is observed that the baseline is very prone to false negatives, i.e., it tends to reject examples from KK classes, as derived from its low $ACC_{KK}$ value. In contrast, the proposed autoencoder-based approaches improve considerably the performance in all cases, discerning more easily KK classes from the unwanted ones.

A similar behavior is observed when looking at the results from the first five trios (from trio 0 to 4). The unsupervised autoencoder shows better performance than the semi-supervised autoencoder with few samples in training. When the number of training samples is four, the semi-supervised always shows the best result. This may reflect that using classification error in the autoencoder training may only have a relevant effect for a sufficient number of shots. Trios 5 and 6 show better results with the semi-supervised autoencoder, especially for two and four shots. Finally, trio 7 shows a quite different behavior, since the unsupervised autoencoder provides the best results for any number of shots. This may be due to the closeness in the feature space of classes 7 and 20.

Regarding the analysis of trios with $O^{*}=0.13$ in Table 5, we can see that the semi-supervised autoencoder obtains better performance in most cases. In this case, where not all unwanted classes are seen in training, semi-supervision helps to obtain more discriminative representations even with few samples. Note, however, that in this set of experiments, the baseline obtains the best result in some cases, like in trios 4, 6 or in all the shots of the trio 7.

Comparing the results obtained in this section with those for the full set with the 24 KK classes, a similar behavior is observed. When $O^{*}=0$ the system is more prone to false negatives, showing lower $ACC_{KK}$ with $O^{*}=0$ than with $O^{*}=0.13$. On the other hand, $ACC_{KU}$ and $ACC_{KUU}$ show worse performance with $O^{*}=0.13$ than with $O^{*}=0$.

With respect to the performance concerning unwanted classes, Table 6 presents the analysis of $ACC_{KUU}$ and $ACC_{UU}$ for the different trio configurations. Note that the former takes into account both unwanted classes seen in training and those that are not. The second only corresponds to the accuracy of the unwanted classes that are only seen in the test stage. The OSR system is expected to have good generalization properties if both are similar. Thus, a more realistic behavior would usually result in a slightly lower metric in $ACC_{UU}$. It is observed that the accuracies are a little lower for autoencoders than for the baseline. Note, however, that this lower performance is significantly compensated by the tradeoff involving better accuracy in KK classes. This means that, although the baseline may have better ability to reject unwanted classes, it is at the expense of rejecting as well pattern sounds. Both the unsupervised and semi-supervised autoencoders show good rejection generalization. Thus, these solutions can be competitive in OSR problems as long as some of the classes to be rejected take part in the training stage.

### 5.3. Performance on ASC Task

Finally, to study the generalization capability of the proposed framework to other tasks not related to the detection of specific sound patterns, we consider Task 1C of the DCASE 2019 [47] edition. This is related to ASC in OSR conditions. The aim of the task is to classify a scene among one of the ten known classes or to consider it as unknown (reject the sample) if it does not belong to any of them. The dataset is designed so that unknown samples are available during training. Therefore, in this task, only the $Acc_{KK}$ and $Acc_{KU}$ metrics are provided. The results are shown in Table 7.

As it can be observed, the results for this task are considerably worse than those for the FSL/OSR dataset. This is because even if there are many samples of a certain class, they are not necessarily very similar or follow a certain spectro–temporal pattern. However, the unsupervised system improves the trade-off of the system proposed as a baseline. It improves considerably the detection of unwanted sounds but worsens the classification of known classes. The semi-supervised system obtains practically the same result of the baseline for the known classes but the detection capability of unwanted sounds is lower. Such result is in line with our expectations. When the framework does not have information about the class it is reconstructing, it tends to create independent internal representations that lead to an improvement in the classification of unwanted ones. On the other hand, when it is forced to obtain representations that do take into account the information of the class, the capability of classifying known classes is improved to the detriment of the detection of unwanted classes.

## 6. Conclusions

This work presented a novel framework capable of classifying audio pattern samples with few data within an open-set recognition context. The proposed system is based on the use of autoencoders to learn latent space representations with few data and a multi-layer perceptron classifier to classify target sound classes and reject unwanted ones. Both unsupervised and semi-supervised autoencoder architectures were considered.

It has been confirmed that, by increasing the number of training samples, a smaller standard deviation and a higher classification accuracy for target classes is obtained, reducing the number of false negatives with respect to the baseline method. In this context, if the number of known known classes is high, the semi-supervised autoencoder seems to perform best. On the other hand, with a small number of known known classes, the autoencoder type has a bigger influence. In this case, the semi-supervised approach usually outperforms the unsupervised one for most openness conditions. Only for zero openness and very few training shots, the unsupervised approach showed increased performance.

## Figures and Tables

**Table 1:** Autoencoder architecture. Values preceded by # correspond to the number of filters and values in parenthesis correspond to kernel size.

Autoencoder Architecture
Dropout(0.1)
Enc. ConvBlock(#8, (3,3))
Enc. ConvBlock(#16,(3,3))
Enc. ConvBlock(#32,(3,3))
Flatten
**Bottleneck**/Dense(128, ‘linear’)
Upsampling
Reshape
Dec. ConvBlock(#32,(3,3))
Dec. ConvBlock(#16,(3,3))
Dec. ConvBlock(#8, (3,3))
Conv2D (#1, (3,3), ‘linear’)

**Table 2:** Number of classes of each configuration and the corresponding openness value.

Pattern Sounds	KK	KU	UU	$T_{\mathrm{TR}}$	$T_{\mathrm{TE}}$	$O^{*}$
		10	0	34	34	0
Full set	24	5	5	29	34	0.04
		0	10	24	34	0.09
		10	0	13	13	0
Trios	3	5	5	8	13	0.13
		0	10	3	13	0.39

**Table 3:** Final classification results (%). Baseline results correspond to the L3 approach framework presented in [22]. The bold numbers indicate winning configurations according to the number of shots.

		Openness Coefficient									
**Shots**	**Framework**	$O^{*}=0$			$O^{*}=0.04$				$O^{*}=0.09$		
		${\mathrm{ACC}}_{\mathrm{KK}}$	${\mathrm{ACC}}_{\mathrm{KU}}$	${\mathrm{ACC}}_{w}$	${\mathrm{ACC}}_{\mathrm{KK}}$	${\mathrm{ACC}}_{\mathrm{KUU}}$	${\mathrm{ACC}}_{\mathrm{UU}}$	${\mathrm{ACC}}_{w}$	${\mathrm{ACC}}_{\mathrm{KK}}$	${\mathrm{ACC}}_{\mathrm{UU}}$	${\mathrm{ACC}}_{w}$
	Baseline	13.8 ± 12.9	99.8 ± 1.0	56.8	57.7 ± 8.4	90.4 ± 5.4	84.8 ± 9.8	74.1	60.1 ± 7.8	39.6 ± 13.4	49.9
1	Unsupervised	68.8 ± 10.3	95.4 ± 3.4	82.1	**76.0 ± 7.6**	90.1 ± 7.6	87.6 ± 10.9	**83.1**	**78.5 ± 7.5**	70.6 ± 15.5	**74.5**
	Semi-supervised	**73.5 ± 8.8**	97.4 ± 2.8	**85.4**	73.1 ± 10.3	90.2 ± 7.4	86.9 ± 11.1	81.7	77.2 ± 9.7	69.7 ± 10.4	73.5
	Baseline	81.1 ± 5.5	99.4 ± 0.8	90.3	83.2 ± 4.8	90.2 ± 5.1	82.5 ± 9.6	86.7	83.3 ± 5.6	33.3 ± 11.6	58.3
2	Unsupervised	82.4 ± 7.2	94.6 ± 3.2	88.5	86.0 ± 5.9	88.7 ± 6.3	84.4 ± 10.0	87.3	86.3 ± 6.6	59.3 ± 14.7	72.8
	Semi-supervised	**90.2 ± 4.9**	98.6 ± 1.8	**94.4**	**89.9 ± 4.4**	93.4 ± 6.0	90.1 ± 9.5	**91.6**	**90.7 ± 4.5**	72.5 ± 8.6	**81.6**
	Baseline	94.8 ± 2.2	99.6 ± 0.4	97.2	94.3 ± 2.2	88.3 ± 5.7	79.4 ± 9.5	91.3	94.8 ± 2.4	26.1 ± 10.1	60.5
4	Unsupervised	91.8 ± 3.8	94.6 ± 2.3	93.2	92.4 ± 4.4	85.8 ± 7.8	80.2 ± 12.0	89.1	91.1 ± 4.9	51.4 ± 17.1	71.2
	Semi-supervised	**97.7 ± 1.6**	99.7 ± 0.5	**98.7**	**97.7 ± 1.5**	97.0 ± 3.1	95.0 ± 5.7	**97.3**	**97.93 ± 1.3**	69.9 ± 8.7	**83.9**

**Table 4:** Results with trios configuration and $O^{*}=0$. The number list under the trio number corresponds to the number of patterns that make up that trio. The bold numbers indicate winning configurations according to the number of shots.

		Framework								
Trio	Shots	Baseline			Unsupervised			Semi-Supervised		
		${\mathrm{ACC}}_{\mathrm{KK}}$	${\mathrm{ACC}}_{\mathrm{KU}}$	${\mathrm{ACC}}_{w}$	${\mathrm{ACC}}_{\mathrm{KK}}$	${\mathrm{ACC}}_{\mathrm{KU}}$	${\mathrm{ACC}}_{w}$	${\mathrm{ACC}}_{\mathrm{KK}}$	${\mathrm{ACC}}_{\mathrm{KU}}$	${\mathrm{ACC}}_{w}$
	1	65.1 ± 16.1	99.4 ± 1.1	82.3	97.4 ± 5.4	97.4 ± 2.5	**97.4**	91.5 ± 10.1	97.9 ± 2.2	94.7
0	2	80.2 ± 15.0	99.6 ± 0.5	89.9	98.4 ± 3.3	98.3 ± 1.5	**98.4**	96.2 ± 6.8	98.2 ± 2	97.2
(1, 9, 17)	4	90.1 ± 14.5	99.7 ± 0.4	94.9	99.0 ± 2.2	98.9 ± 1.0	98.9	98.8 ± 3.7	99.4 ± 0.7	**99.1**
	1	68.9 ± 12.9	99.9 ± 0.2	84.4	96.9 ± 6.4	96.2 ± 3.8	**96.6**	94.8 ± 9.3	96.8 ± 3.7	95.8
1	2	84.7 ± 16.5	99.9 ± 0.4	92.3	99.0 ± 1.3	98.5 ± 1.5	96.8	98.4 ± 3.4	98.3 ± 2.1	**98.4**
(10, 12, 19)	4	88.0 ± 15.6	99.9 ± 0.4	93.9	99.5 ± 0.9	98.9 ± 1.2	99.2	99.2 ± 1.8	99.4 ± 1.1	**99.3**
	1	55.5 ± 18.6	99.9 ± 1.0	77.7	92.1 ± 8.5	97.4 ± 3.1	**94.8**	86.4 ± 12.2	98.4 ± 2.9	92.4
2	2	76.1 ± 14.7	99.9 ± 0.1	88.0	95.9 ± 6.7	98.6 ± 1.6	**97.3**	94.8 ± 6.2	99.1 ± 1.3	97.0
(2, 14, 22)	4	83.1 ± 20.7	99.9 ± 0.1	91.5	97.8 ± 2.3	98.8 ± 1	98.3	98.6 ± 2.2	99.8 ± 0.8	**99.2**
	1	53 ± 12.1	99.9 ± 0.4	76.5	90.0 ± 11.4	94.7 ± 3.7	**92.3**	83.5 ± 12.2	95.2 ± 3.6	89.3
3	2	64.6 ± 16.1	99.9 ± 0.3	82.2	93.8 ± 7.2	96.8 ± 2.2	**95.3**	89.8 ± 10.7	96.9 ± 2.4	93.4
(3, 6, 13)	4	77.4 ± 19.0	99.8 ± 0.9	88.6	96.9 ± 5.5	97.9 ± 1.4	97.4	97.1 ± 3.9	99.0 ± 1.1	**98.0**
	1	71.7 ± 15.2	100 ± 0	85.8	91.2 ± 9.3	96.1 ± 3.1	**93.7**	87.3 ± 12.2	96.3 ± 3.2	91.8
4	2	86.8 ± 14.5	100 ± 0	93.4	94.7 ± 6.3	97.8 ± 1.7	**95.3**	93.2 ± 8.4	97.3 ± 2.1	**95.3**
(4, 5, 16)	4	88.1 ± 18.6	99.9 ± 0.6	94.0	96.3 ± 4.8	98.4 ± 1.5	97.4	99.1 ± 1.9	99.2 ± 1.0	**99.1**
	1	76.5 ± 15.2	99.9 ± 0.2	88.2	92.3 ± 9.7	98.0 ± 2.3	95.2	92.4 ± 8.5	98.5 ± 2.6	**95.4**
5	2	85.1 ± 15.4	99.9 ± 0.1	92.5	95.4 ± 6.8	98.8 ± 1.6	97.1	96.0 ± 5.2	99.2 ± 1.2	**97.6**
(18, 21, 23)	4	89.3 ± 16.4	100 ± 0.1	94.6	98.0 ± 2.6	99.3 ± 0.9	98.6	99.0 ± 1.4	99.8 ± 0.3	**99.4**
	1	87.0 ± 13.5	99.7 ± 0.5	93.4	95.3 ± 7.2	97.0 ± 3.1	96.1	94.4 ± 7.7	97.9 ± 2.7	**96.2**
6	2	87.6 ± 16.0	99.6 ± 0.6	93.6	96.2 ± 4.9	97.9 ± 1.9	97.1	97.1 ± 4.3	98.8 ± 1.5	**98.0**
(8, 11, 24)	4	89.9 ± 14.5	99.7 ± 0.5	94.8	99.1 ± 1.9	98.8 ± 1.2	98.9	99.3 ± 1.4	99.6 ± 0.6	**99.4**
	1	66.4 ± 15.7	99.6 ± 0.6	83.0	89.0 ± 9.7	96.3 ± 3.0	**92.7**	77.4 ± 14.2	97.2 ± 3.1	87.3
7	2	82.1 ± 13.7	99.5 ± 0.7	90.8	92.8 ± 6.4	97.8 ± 1.8	**95.3**	86.4 ± 9.2	98.2 ± 1.9	92.3
(7, 15, 20)	4	83.7 ± 15.3	99.5 ± 0.9	91.6	95.5 ± 3.9	98.8 ± 1.1	**97.2**	91.0 ± 7.3	99.1 ± 1.1	95.1

**Table 5:** Results with trios configuration and $O^{*}=0.13$. The number list under the trio number corresponds to the number of patterns that make up that trio. The bold numbers indicate winning configurations according to the number of shots.

		Framework								
Trio	Shots	Baseline			Unsupervised			Semi-Supervised		
		${\mathrm{ACC}}_{\mathrm{KK}}$	${\mathrm{ACC}}_{\mathrm{KU}}$	${\mathrm{ACC}}_{w}$	${\mathrm{ACC}}_{\mathrm{KK}}$	${\mathrm{ACC}}_{\mathrm{KU}}$	${\mathrm{ACC}}_{w}$	${\mathrm{ACC}}_{\mathrm{KK}}$	${\mathrm{ACC}}_{\mathrm{KUU}}$	${\mathrm{ACC}}_{w}$
	1	85.88 ± 13.4	97.7 ± 4.6	91.8	97.7 ± 5.2	93.9 ± 5.7	**95.8**	95.4 ± 7.4	93.9 ± 6.6	94.6
0	2	89.2 ± 12.5	99.6 ± 0.5	94.4	99.1 ± 1.8	96.5 ± 3.7	**97.8**	97.7 ± 4.7	96.9 ± 3.7	97.3
(1, 9, 17)	4	97.5 ± 8.1	99.7 ± 0.4	98.6	99.4 ± 1.8	98.1 ± 2.5	98.7	99.2 ± 2.1	98.4 ± 1.9	**98.8**
	1	88.8 ± 13.1	98.3 ± 2.8	93.5	98.5 ± 3.7	88.6 ± 9.4	93.6	97.6 ± 6.2	89.7 ± 9.8	**93.7**
1	2	89.0 ± 14.5	98.7 ± 2.4	93.8	99.4 ± 1.2	94.7 ± 5.8	97.0	98.6 ± 2.7	95.6 ± 4.7	**97.2**
(10, 12, 19)	4	96.2 ± 9.6	96.7 ± 3.1	96.5	99.5 ± 1.0	97.7 ± 3.0	**99.6**	99.4 ± 1.2	97.7 ± 4.4	98.6
	1	78.4 ± 13.4	99.8 ± 0.9	89.1	95.2 ± 7.3	89.6 ± 9.0	92.4	94.1 ± 8.3	94.6 ± 6.4	**94.3**
2	2	82.6 ± 13.9	99.8 ± 0.5	91.2	97.6 ± 4.2	93.2 ± 7.1	95.4	97.7 ± 3.8	97.1 ± 5.1	**97.4**
(2, 14, 22)	4	91.9 ± 12.3	99.4 ± 0.9	95.6	98.9 ± 2.0	94.1 ± 6.9	96.5	98.6 ± 3.0	99.1 ± 1.8	**98.9**
	1	72.3 ± 13.4	96.2 ± 4.2	84.3	94.2 ± 8.2	86.0 ± 8.7	**90.1**	90.4 ± 10.9	88.7 ± 8.3	89.6
3	2	78.37 ± 13.7	95.7 ± 4.6	87.2	96.9 ± 4.3	90.0 ± 7.9	93.5	94.8 ± 7.4	94.0 ± 5.9	**94.4**
(3, 6, 13)	4	90.3 ± 11.4	92.0 ± 3.2	91.1	97.1 ± 3.9	95.2 ± 4.6	96.1	97.0 ± 4.9	96.6 ± 3.6	**96.8**
	1	88.5 ± 10.1	99.3 ± 1.3	**93.9**	94.6 ± 7.4	91.3 ± 6.7	92.9	94.2 ± 8.6	89.1 ± 9.9	91.6
4	2	93.2 ± 9.2	99.4 ± 1.1	**96.3**	96.2 ± 5.5	95.1 ± 4.8	95.6	97.1 ± 5.5	94.2 ± 5.8	95.7
(4, 5, 16)	4	97.0 ± 9.1	99.0 ± 1.2	98.0	98.2 ± 3.0	96.2 ± 3.8	97.2	99.1 ± 2.4	97.6 ± 2.9	**98.4**
	1	87.9 ± 11.8	99.1 ± 1.2	93.5	96.2 ± 5.2	93.2 ± 6.1	94.7	95.9 ± 7.0	95.5 ± 6.2	**95.7**
5	2	93.4 ± 7.7	98.8 ± 1.2	96.1	98.0 ± 2.6	95.9 ± 4.0	97.0	98.8 ± 2.0	98.6 ± 2.9	**98.7**
(18, 21, 23)	4	97.2 ± 8.1	98.3 ± 1.2	97.7	98.8 ± 1.9	94.0 ± 5.9	96.4	99.6 ± 1.0	99.7 ± 0.6	**99.6**
	1	96.0 ± 7.8	99.3 ± 0.8	**97.6**	96.4 ± 6.6	93.9 ± 5.07	95.1	97.00 ± 5.73	94.23 ± 6.35	95.6
6	2	95.8 ± 9.1	99.4 ± 0.7	97.6	98.6 ± 3.1	96.0 ± 3.9	97.3	99.0 ± 2.7	97.2 ± 3.9	**98.1**
(8, 11, 24)	4	96.8 ± 9.2	99.2 ± 0.8	98.0	99.5 ± 1.0	95.9 ± 4.4	97.7	99.5 ± 1.2	98.7 ± 2.1	**99.1**
	1	87.0 ± 11.4	97.6 ± 2.9	**92.3**	91.1 ± 9.2	87.3 ± 7.8	89.2	84.9 ± 12.4	87.3 ± 8.9	86.1
7	2	90.0 ± 9.8	98.6 ± 1.7	**94.3**	94.8 ± 6.0	92.4 ± 5.6	93.6	89.2 ± 9.4	93.0 ± 6.2	91.1
(7, 15, 20)	4	94.4 ± 10.1	98.5 ± 1.5	**96.5**	96.0 ± 4.2	95.2 ± 4.2	95.6	93.8 ± 5.8	95.8 ± 4.2	94.8

**Table 6:** Results of unwanted category with trios configuration and $O^{*}=0.13$. The number list under the trio number corresponds to the number of patterns that make up that trio.

		Framework					
Trio	Shots	Baseline		Unsupervised		Semi-Supervised	
		${\mathrm{ACC}}_{\mathrm{KUU}}$	${\mathrm{ACC}}_{\mathrm{UU}}$	${\mathrm{ACC}}_{\mathrm{KUU}}$	${\mathrm{ACC}}_{\mathrm{UU}}$	${\mathrm{ACC}}_{\mathrm{KUU}}$	${\mathrm{ACC}}_{\mathrm{UU}}$
	1	97.7 ± 4.6	98.4 ± 4.1	93.9 ± 5.7	93.7 ± 8.8	93.9 ± 6.6	93.0 ± 9.2
0	2	99.6 ± 0.5	99.8 ± 0.6	96.5 ± 3.7	96.2 ± 5.6	96.9 ± 3.7	96.2 ± 6.5
(1, 9, 17)	4	99.7 ± 0.4	99.9 ± 0.4	98.1 ± 2.5	98.2 ± 3.7	98.4 ± 1.9	98.5 ± 2.8
	1	98.3 ± 2.8	96.8 ± 5.6	88.6 ± 9.4	87.0 ± 12.5	89.7 ± 9.8	87.0 ± 13.5
1	2	98.7 ± 2.4	97.6 ± 4.7	94.7 ± 5.9	93.4 ± 8.9	95.6 ± 4.7	94.4 ± 7.1
(10, 12, 19)	4	96.7 ± 3.1	93.8 ± 5.8	97.7 ± 3.0	96.8 ± 4.6	97.7 ± 4.4	96.6 ± 6.8
	1	99.8 ± 0.9	99.7 ± 1.7	89.6 ± 8.9	85.3 ± 13.9	94.6 ± 6.4	92.2 ± 9.7
2	2	99.8 ± 0.5	99.7 ± 0.6	93.2 ± 7.1	89.2 ± 11.7	97.1 ± 5.1	95.9 ± 7.7
(2, 14, 22)	4	99.4 ± 0.9	99.0 ± 1.5	94.1 ± 6.9	90.2 ± 12.3	99.1 ± 1.8	98.7 ± 3.3
	1	96.2 ± 4.2	92.7 ± 8.2	86.0 ± 8.7	84.7 ± 13.4	88.7 ± 8.3	87.5 ± 12.6
3	2	95.7 ± 4.6	91.6 ± 8.7	90.0 ± 7.9	87.5 ± 13.3	94.0 ± 5.9	93.3 ± 9.5
(3, 6, 13)	4	92.0 ± 3.2	84.8 ± 6.0	95.2 ± 4.6	93.5 ± 7.3	96.6 ± 3.6	96.0 ± 5.7
	1	99.3 ± 1.3	98.6 ± 2.5	91.3 ± 6.7	90.4 ± 9.5	89.1 ± 9.9	87.0 ± 13.6
4	2	99.4 ± 1.1	98.8 ± 2.2	95.1 ± 4.8	94.4 ± 7.1	94.2 ± 5.8	92.5 ± 9.8
(4, 5, 16)	4	99.0 ± 1.2	98.1 ± 2.2	96.2 ± 3.8	94.6 ± 6.6	97.7 ± 2.9	97.2 ± 4.7
	1	99.1 ± 1.2	98.5 ± 2.2	93.2 ± 6.1	90.0 ± 9.7	95.5 ± 6.3	94.0 ± 9.0
5	2	98.8 ± 1.2	97.8 ± 2.3	95.9 ± 4.0	93.5 ± 6.9	98.6 ± 2.9	97.8 ± 5.1
(18, 21, 23)	4	98.3 ± 1.2	96.8 ± 2.1	94.0 ± 5.9	98.4 ± 10.9	99.7 ± 0.6	99.4 ± 1.2
	1	99.3 ± 0.8	99.4 ± 0.6	93.9 ± 5.1	92.5 ± 7.9	94.2 ± 6.4	93.4 ± 8.9
6	2	99.4 ± 0.7	99.2 ± 1.0	96.0 ± 3.9	94.0 ± 7.0	97.2 ± 3.9	96.4 ± 6.8
(8, 11, 24)	4	99.2 ± 0.8	98.9 ± 1.0	95.9 ± 4.4	93.1 ± 8.0	98.7 ± 2.1	98.2 ± 3.0
	1	97.6 ± 2.9	96.8 ± 5.4	87.3 ± 7.8	83.7 ± 12.1	87.3 ± 8.9	82.6 ± 13.6
7	2	98.6 ± 1.7	98.4 ± 3.0	92.4 ± 5.6	89.7 ± 9.3	93.0 ± 6.2	89.6 ± 9.9
(7, 15, 20)	4	98.5 ± 1.5	98.1 ± 2.7	95.2 ± 4.2	93.1 ± 7.6	95.8 ± 4.2	93.4 ± 7.4

**Table 7:** Results(%) of the proposed frameworks using DCASE (Detection and Classification of Acoustic Scenes and Events) 2019 Task 1C dataset. The baseline results correspond to the one presented by the task organization as a starting point.

Framework	${\mathrm{Acc}}_{\mathrm{KK}}$	${\mathrm{Acc}}_{\mathrm{KU}}$	${\mathrm{Acc}}_{w}$
Baseline	54.2	43.1	48.7
Unsupervised	39.3	69.0	54.1
Semi-supervised	53.5	25.8	39.6
